# The Use of Brewer’s Spent Grain after Beer Production for Energy Purposes

**DOI:** 10.3390/ma15103703

**Published:** 2022-05-22

**Authors:** Szymon Głowacki, Agnieszka Salamon, Mariusz Sojak, Weronika Tulej, Andrzej Bryś, Taras Hutsol, Marek Salamon, Savelii Kukharets, Monika Janaszek-Mańkowska

**Affiliations:** 1 Institute of Mechanical Engineering, Warsaw University of Life Sciences, 02-787 Warsaw, Poland; mariusz_sojak@sggw.edu.pl (M.S.); andrzej_brys@sggw.edu.pl (A.B.); monika_janaszek@sggw.edu.pl (M.J.-M.); 2 Institute of Agricultural and Food Biotechnology–State Research Institute, 02-532 Warsaw, Poland; agnieszka.salamon@ibprs.pl; 3 Department of Mechanics and Agroecosystems Engineering, Polissia National University, 10008 Zhytomyr, Ukraine or pro-gp@pdatu.edu.ua (T.H.); saveliy_76@ukr.net (S.K.); 4 Faculty of Manufacturing Engineering, Warsaw University of Life Sciences, 02-787 Warsaw, Poland; mareksal93@gmail.com

**Keywords:** energy, biomass, renewable resources

## Abstract

The aim of this study was to assess the possibilities to use brewer’s spent grains (BSGs) left over from beer production for energy purposes, and to determine its calorific value and chemical composition. The research materials were samples of wet spent grain from a brewery in Poland. Three samples, that are different in ingredient composition, were examined. The examined samples of BSGs were characterised by humidity that is typical for this product (approx. 77–80%). Convective drying of the spent grain contributed to a reduction in the water content in the biomass to below 10%. Samples of dry spent grain that were examined contained a similar amount of ash (3.8–4.1% d.m.) and organic matter (91.0–91.9% d.m.). All the examined spent grain samples demonstrated similar volatile matter content—approx. 77.8–78.7% d.m. and calorific value—approx. 15.6–15.9 MJ/kg. The estimated calorific value for wet samples (approx. 1.4–2.0 MJ/kg) indicated that it is necessary to lower water content in the biomass in order to improve its energy properties.

## 1. Introduction

A waste product of beer production is a large volume of biomass. Its rational use does not only contribute to natural environment protection but may also bring notable benefits to the brewery and other fields of the industry. Brewer’s spent grain (BSGs), which is a perishable product due to high water content, is produced in largest quantities.

In Poland, beer production from malt amounted to approx. 38.4 million hL in 2020 [1]. With the assumption that for 1 hL of beer produced, 20 kg of wet BSG is left over [2,3,4], Polish breweries generate approx. 0.74 million tons of this waste product. In the European Union countries, the average annual production of BSG amounts to approx. 3.4 million tons, and the global production amounts to approx. 39 million tons [5,6].

The global energy crisis and greenhouse effect are stimuli for searching for renewable resources of energy. Legal regulations adopted in the European Union make the member countries limit the emission of greenhouse gases by obtaining energy from natural renewable resources such as solar energy, wind, and water, and try to find new renewable resources with no negative impact on the environment. Biomass is one of the most promising sources of renewable energy. Plant-derived biomass is produced in the largest quantities and may be used for energy purposes by direct combustion or processing into liquid or gaseous fuels. BSG left over from the production of beer is a valuable raw material, which may be used for energy purposes. Its availability and low price make its use in the energy industry justified, taking into consideration the fact that interest in BSG as animal feed has decreased. Obtaining renewable energy from BSG is predominantly based on biogas and bioethanol production, as well as thermochemical conversion.

## 2. Literature Review

### 2.1. Beer Production and Biomass Left over from Beer Production

Brewing is one of the largest branches of the food industry, with beer being one of the most popular alcoholic beverages consumed worldwide, including Poland. The production of beer from malt amounted to approx. 38.4 million hL in 2020 [1,7].

Rational use of biomass may both be profitable to the brewery and contribute to natural environment protection. 

Brewer’s spent grain (BSG), left over from the production of beer in the brewery, is the biomass with the highest weight. During the stage of mashing the malt, enzymatic changes occur, which result in approximately 60–70% of dry mass being transferred to malt mash (wort), and the rest remaining in the BSG. Malts used for the production of beer may be produced from different grains, although the basic grains are produced from brewer’s barley. Malts obtained from other cereal grains, e.g., wheat, are also used for the production of beer. In the technological process of beer brewing, it is acceptable to replace some malt with unmalted grain material, e.g., barley. The maximum amount of unmalted material is 45% of the total raw material for beer production [8]. Brewing is followed by filtration of brewer’s mash and lautering the spent grain with hot water (approx. 78 °C). Finally, spent grain is removed from the lauter tub or filter press, transported to the storage bin using the screw conveyor, and then to the distribution tank outside the brewery. Depending on the method of BSG sparging, its moisture ranges between 75 and 85% [2,4,9,10,11,12].

Wet BSG has a consistency of thickly ground malt grain, with a light brown to brown colour, and a sweet taste and smell are typical for materials that are used for the production of beer. It consists of undissolved malt particles, mostly of husks of malt grains mixed with some endosperm and tegument, non-saccharified starch, and other substances, which precipitated during the mashing. Chemical composition of BSG may vary considerably, depending on various factors, including barley variety, harvest time, conditions of barley cultivation, malting and mashing parameters, the amount and type of unmalted raw materials in a batch, or the degree of sparging the wort extract from the malt. 

### 2.2. Directions of Brewer’s Spent Grain (BSG) Use

Relatively high water content (approx. 75–85%) remains of carbohydrates and proteins in brewer’s spent grain (BSG) make it a perishable product which rots quickly in unfavourable ambient conditions, in particular, temperatures favourable for the development of harmful microorganisms [2,4,9,11,13,14].

The main direction of use of BSG left over from the production of beer is using it as animal feed. Breweries sell spent grain to local farmers. The problem with selling BSG may be encountered during spring and summer as the farmers’ interest in purchasing spent grain decreases due to higher availability of cheaper green fodder. Nevertheless, this waste product is the subject of interest in different areas due to its low price, all-year-round availability, and valuable chemical composition [5,15,16].

Directions of brewer’s spent grain (BSG) use, apart from using it as a fodder, include food and energy production as well as other biotechnological processes [16,17]. Few studies have focused on the use of BSG for the production of building materials. Russ et al. [18] examined technical properties of bricks that contained BSG as a component which increased their porosity. Low ash content and high content of fibre (lignin, hemicellulose, and cellulose) made the authors of the above-mentioned study examine properties of BSG and assess its usefulness as a building material. The results obtained by the researchers indicated that the finished bricks containing the addition of BSG had higher resistance and porosity as well as smaller density after firing, which improved their thermo-insulating conditions in comparison with the bricks produced from clay.

### 2.3. Generation of Energy from Brewer’s Spent Grain (BSG) 

The worldwide energy crisis is a stimulus for searching for new sources of renewable energy. High availability of BSG and its low price make it challenging to use it in the power industry. This product, due to its high content of water (approx. 80%), protein, and ash, is not a material that can be easily used in this area. The elemental composition of spent grain (carbon—46.8%, hydrogen—7.0%, oxygen—38.7%, nitrogen—0.7%, and sulphur—0.3%) does not meet the standards for fossil fuels [19]. Similar results of elemental analysis were obtained by other researchers [20,21,22,23], except for the amount of nitrogen, which was at the level of 3.46–5.45%. The discussed direction of spent grain use may not cover the total demand of the brewery for energy but it may be a stimulus for the introduction of a 100% renewable energy plan in a brewery. Obtaining energy from BSG mainly involves production of biogas and bioethanol and thermochemical conversion.

#### 2.3.1. Biogas Production

Biomass from farming and the food industry is a valuable raw material for the production of biogas, which is a renewable resource of energy. Biogas is produced in the process of methane fermentation of organic matter contained in substrates of plant and animal origin (BSG, beet pulp, pressed fruit pomace, maize silage, cattle manure, liquid manure, post-slaughter waste, etc.) [24,25]. Biogas mainly consists of methane (CH_4_—approx. 60%) and carbon dioxide (CO_2_—approx. 40%), with trace amounts of hydrogen sulphide (H_2_S), hydrogen (H_2_), nitrogen (N_2_), carbon oxide (CO), oxygen (O_2_), steam (H_2_O), and other gases [26,27,28]. Biogas production in the process of anaerobic fermentation consists of two stages, namely: initial hydrolysis of raw material to increase availability of components and the methane-forming stage during which acid-forming microorganisms metabolize substances released during the hydrolysis stage to short-chain fatty acids such as acetic acid, butyric acid, and propionic acid. The acids are then transformed into methane by methane bacteria. Hydrolysis of the raw material may involve chemical-thermal treatment, size reduction (milling, cutting) or treatment with enzymes, decomposing lignocellulose fractions. The main goal of this stage is decomposition of complex organic compounds into less complex compounds in order to facilitate their availability for methane bacteria growth [27,28,29].

Brewer’s spent grain (BSG) use for the production of biogas involves obtaining heat energy for the brewery [3,4,27]. Based on the data in the literature, Mussatto [27] wrote that following a 15-day anaerobic fermentation process of the BSG in laboratory conditions, approx. 3476 cm^3^ of biogas was obtained from 100 g of spent grain. Based on this experiment, theoretical methane production efficiency was estimated at approx. 96 m^3^ per 1 ton of spent grain.

Improvement of energy properties of BSG requires pre-treatment of the raw material, which has been emphasised by researchers [3,4,10]. Application of mechanical pressing of wet spent grain using a screw press by Weger et al. [3] made it possible to obtain a solid fraction in the form of thick pulp, which mostly consists of combustible components, and to separate a liquid fraction, rich in dissolved nitrogen compounds. As a result of mechanical pressing of the wet raw material, approx. 43.3% of the solid fraction and approx. 56.7% of the liquid fraction were obtained, which amounted to 162 kg d.m. of solid substance (thick pulp) and 32 kg d.m. of liquid, respectively. The obtained fractions of the BSG were used for the brewery’s energy needs.

Čater et al. [29] investigated the addition of anaerobic hydrolytic bacteria to BSG and its effect on biogas production. Pure cultures or consortia of bacteria were used to increase the hydrolysis of lignocellulose compounds in the spent grain and to produce biogas. The increase in methane production was possible by enriching the substrate with the monoculture of Pseudobutyrivibrio xylanivorans (+17.8%), followed by the complex of Pseudobutyrivibrio xylanivorans and Fibrobacter succinogenes (+6.9%), as well as Clostridium cellulovorans and Fibrobacter succinogenes (+4.9%).

According to the study by Lewicki et al. [24] conducted in the farm biogas plant, the amount of biogas and methane obtained from wet spent grain was 135 and 80 m^3^/t of fresh mass, respectively. Methane share in biogas amounted to 54.4% and was comparable to beet pulp (54.1%). The highest yield of biogas and methane per 1 ton was obtained from slaughterhouse waste (461 and 303 m^3^/t, respectively).

The production of biogas from brewer’s spent grain, wheat waste, and rice husks was studied by Ezekoye et al. [26]. In order to increase biochemical reactions during fermentation of waste and increase methane efficiency, catalysts such as clay, limestone, and wood ash were used. The results showed that the production of biogas for the mix of spent grain and clay occurred during the fourth 24 h day, with the total volume amounting to 69.3 dm^3^. Mixed biogas (wheat waste and ash) was obtained during the thirty-fourth 24 h day, and at the end of the fermentation (60 days), the amount of the obtained biogas was equal to 178.5 dm^3^. The analysis of the composition showed that the share of methane in biogas for the above-mentioned variants amounted to 75.6 and 81.7%, respectively.

#### 2.3.2. Production of Bioethanol

Interest in bioethanol production observed recently largely has resulted from the increase in prices of crude oil, climatic changes, and search for renewable resources of energy. Bioethanol is an alternative to petrol, efficient to produce and safe for the natural environment. Permitting the production of bioethanol from lignocellulose waste material has resulted in the consideration of BSG, rich in these substances, as a raw material for such production. These raw materials are mostly composed of cellulose—glucose polymer, and hemicellulose—a mixture of polysaccharides composed of glucose, mannose, xylose, arabinose, and lignin. Production of bioethanol from spent grain requires chemical or enzymatic hydrolysis of the above-mentioned non-starch polysaccharides to fermentable sugars in the process of microbial fermentation [15,27,30,31,32].

The study by Wilkinson et al. [31] aimed to assess thermal hydrolysis (121 °C, 30 min) in an acidic or alkaline environment as the initial stage of BSG treatment for the production of bioethanol. It was demonstrated that the most effective catalytic agent of the saccharification reaction was a solution of hydrochloric acid (HCl), which allowed for glucose productivity at the level of 86%. According to subsequent research conducted by the authors [32], the combined use of Aspergillus oryzae and Saccharomyces cerevisiae resulted in highest productivity of bioethanol obtained from spent grain (37 g/L of ethanol after ten 24 h days of fermentation), in comparison with other assessed microorganism groups. Based on the results, it was estimated that in the process of fermentation of 1 ton d.m. of spent grain mixed with 36 hL of water, 94 kg of pure bioethanol can be obtained, and the process required neither pre-treatment nor the addition of cytolitic enzymes. Other researchers [30] used the mixed culture of Fusarium oxysporum and Saccharomyces cerevisiae as a source of enzymes for simultaneous saccharification and fermentation of BSG for the production of bioethanol. The productivity of ethanol equal to 57% was obtained using the mixed culture, and it was higher than the amount of ethanol obtained using single cultures of F. oxysporum (26%) and S. cerevisiae (33%) in the same conditions of fermentation. 

#### 2.3.3. Thermochemical Conversion

One method of obtaining energy from biomass, i.a., brewer’s spent grain (BSG), is using thermochemical conversion technologies such as pyrolysis and combustion. 

The calorific value of BSG amounts to approx. 18–20 MJ/kg d.m. [10,18], which makes it an interesting raw material for the production of energy in the process of combustion. On the other hand, due to the high water content (approx. 80%), BSG has low calorific value, and thermal drying is not often economically cost-effective. Therefore, in order to improve its combustible properties, mechanical pre-treatment using presses to dewater the spent grain seems indispensable. Stable conditions of spent grain combustion require a water content below 55% [3,4]. Heat produced during combustion may, to a certain extent, be a supplementary source of energy that can be used by the brewery. 

Research on using spent grain as a source of energy for the brewery was conducted by Zanker and Kepplinger [4]. They formulated technical assumptions and implemented a complex system for BSG processing, allowing for efficient use of the generated heat power by the brewery. The technology involved mechanical pre-treatment of spent grain, i.e., pressing it to reduce the water content to approx. 40–50%. This allowed for obtaining a biologically stable material, which could be deposed for a period of time in the buffer tank. ‘Dried’ waste was combusted in a biomass boiler, which supplied approx. 60% of primary energy in the form of steam. Ash was used as a fertilizer component due to its chemical composition. A project that was proposed by Bruijn et al. [10] combined the environmentally friendly method of use of nutritious values of the biomass waste, produced by the brewery with the production of energy from the biomass. Wet BSG was separated into two fractions using the developed separating system, which consisted of a tank for mixing the spent grain with the recirculatory liquid, an apparatus in which coagulation forces separated protein from husks, expeller for size-reduced spent grain, liquid fraction decanter, etc. The process was to reduce the content of nitrogen in the dewatered spent grain in order to minimize the emission of nitrogen oxides during combustion and to maximize the content of protein in the obtained liquid fraction. The dewatered spent grain with the dry mass content above 40% was combusted in the steam biomass boiler, and the obtained energy covered approx. 30% of the demand of the brewery for fossil fuels. The analysis of energy efficiency demonstrated that the calorific value of dewatered spent grain amounted to 7.2 MJ/kg, and after conversion to dry mass—45%.

Manyuchi and Frank [33] conducted research on spent grain use for the production of electrical power, using direct combustion technology. The calorific value of the raw material was determined at the medium level of approx. 12.6 MJ/kg. After initial drying of spent grain (approx. 6% of moisture), it was fed into the biomass boiler with the speed of 1100 kg/h, which produced approx. 1700 kg of steam with a temperature of approx. 300 °C and pressure of 9 bars per hour. Overheated steam was converted into inductive energy in the generator, producing a voltage of 1 MW. Based on this experiment, it was concluded that BSG might be an alternative source of energy for the brewery. 

Another method of obtaining energy from BSG was production of charcoal bricks from this waste material [27]. The process of production comprised the initial stage of spent grain drying and pressing, followed by combustion with limited access of oxygen. Charcoal bricks produced with this method had high calorific value (27 MJ/kg), which was higher than the calorific value of the BSG, and close to the calorific value of charcoal produced from other raw materials, such as wood, sugarcane, grape bagasse, or hazelnut shell. On the other hand, charcoal bricks from BSG demonstrated worse combustible properties than charcoal due to the high ignition temperature and longer combustion time. 

Mahmood et al. [34] studied pyrolysis of brewer’s spent grain. As a result of partial pyrolysis performed at a temperature of 450 °C using a pyroformer, the following were obtained: 29% of coal, 51% of liquid fuel (biooil), and 19% of pyrolitic gases. The obtained fraction of bio-oil was a mixture of hydrocarbons with low and medium molecular mass, such as benzene, cyclotetraoctaene, hexane, toluene, xylene, phenolic, and other aromatic compounds. On the other hand, placing the catalytic reformer downstream of the bench-scale batch reactor and the use of a commercial nickel catalyst for reforming fumes from the pyrolysis of spent grains at temperatures of 500, 750, and 850 °C resulted in a significant increase in the amount of stable gases, mainly (H_2_ and CO) with an H_2_ content exceeding 50% vol. at higher reforming temperatures. Along with the increase in the reforming temperature, a significant decrease in bio-oil efficiency and an increase in the calorific value of the gaseous product in the range of 10.8–25.2 MJ/m^3^ were observed. Ren et al. [35] demonstrated that heterogeneous catalysts, such as nickel-based catalysts, noble metal-based catalysts, natural catalysts, and char catalysts, show high activity against tar removal after biomass gasification, and thus show the production of synthetic gas.

Experiments showing the influence of the carrier gas on the amount and quality of pyrolysis products achieved from the thermal process of BSG were conducted by Bieńek et al. [23]. Three types of carrier gases were investigated (nitrogen, argon, and carbon dioxide) at three temperatures (500, 600, and 700 °C). There were no significant differences in product yields in various atmospheres. At a temperature of 500 °C, the yield changed by 28% and at a temperature of 700 °C—by 19%. Moreover, the char from CO_2_ pyrolysis was about 2% richer in carbon and it had no effect on the combustion properties of the char. The assessed oil fraction was characterised mainly by acids with a maximum content of 68% at 600 °C in an argon atmosphere and the acid concentration depended on the carrier gas as follows: Ar > N_2_ > CO_2_.

## 3. Goal and Scope

The goal of the study was to assess the possibility to use brewer’s spent grain (BSG) for energy purposes and to determine its calorific value and chemical composition, depending on ingredient composition. 

The scope of research comprised:

- Examination of the composition of BSG (content of moisture, ash, and organic matter);
- Determination of the calorific value of BSG with the calorimetric method.

## 4. Methodology and Research Objects

### 4.1. Description of Samples

**Figure 1:**
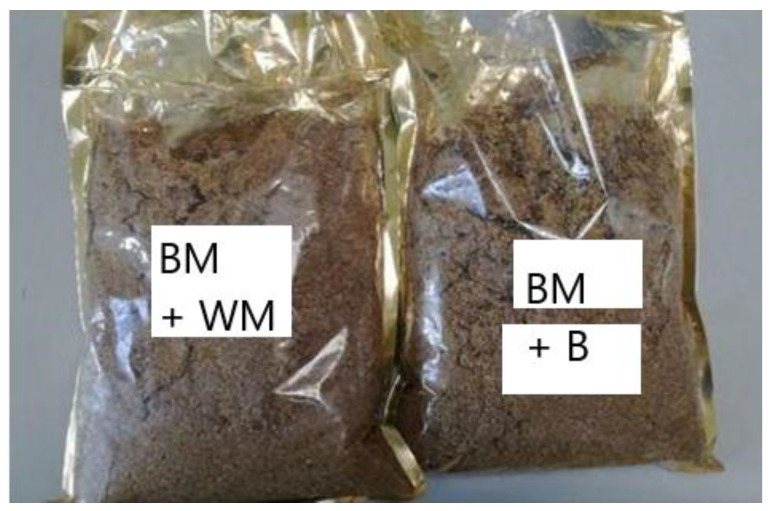
The brewer’s spent grain used for the experiments.

The materials for experiments were samples of BSG (Figure 1) from an industrial brewery in Poland. 

The samples of wet BSG were different in their ingredient composition, i.e., the type and amount of ingredients in the batch for the production of brewer’s wort:

- BSG from barley malt (BM) (100%); sample labelled BM;
- BSG from barley malt (BM) with the addition of hulled barley (B) (unmalted ingredient—up to 45%); sample labelled BM + B;
- BSG from barley malt (BM) and wheat malt (WM) (minimum 50%); sample labelled BM + WM.

### 4.2. Methodology

#### 4.2.1. Determination of Ash Content

Determination of the ash content was performed using the gravimetric method after incineration according to PN-EN ISO 18122:2016-01 [36]. The method consists of incineration of the sample of BSG at a temperature of 900 °C, and gravimetric determination of the organic residue after incineration. 

**Figure 2:**
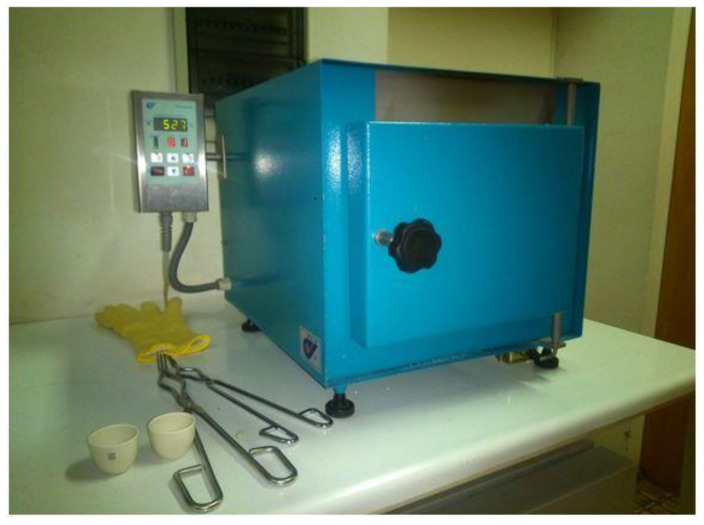
Muffle furnace for the incineration of samples.

Prior to determination of the ash content, the dried porcelain crucible was roasted to a constant mass in the combustion furnace SM-2002 (Figure 2) (Czylok, Jastrzębie-Zdrój, Poland) at a temperature of 900 ± 15 °C for 60 min, then the crucible was placed in the exicator to cool down. 

The empty crucible was weighed on the analytic scales CP224S-OCE (Sartorius, Göttingen, Germany) with the accuracy of 0.0001 g (c), and a sample of approx. 1 g of dry spent grain was placed in it (a). The crucible containing the sample was placed on the electric cooking stove under the ventilating hood, and it was heated carefully until the content of the crucible incinerated. The crucible with the sample was placed in the muffle furnace for combustion, and the temperature was increased in the furnace for 45 min to reach a temperature of 900 °C, and it was then roasted at a temperature of 900 ± 15 °C for 60 min until the colour of ash was uniform. After cooling, the crucible with the ash was weighed on the analytic scales with the accuracy of 0.0001 g (b). The determination was conducted in two replicates.

Ash content (A) (%) was calculated from the formula:

(1) $$A=\frac{\left(b-c\right)\cdot 100}{a-c}$$

where:

a—mass of the crucible with the sample prior to roasting, in grams (g),

b—mass of the crucible with the sample, in grams (g), and

c—mass of the crucible, in grams (g).

#### 4.2.2. Determination of Moisture Content

Determination of the moisture content was performed using the weight method after drying in an oven at 105 °C according to PN-EN ISO 18134-3:2015-11 [37]. The principle of the method consists of drying a weighed sample of spent grain and comparing the weight of the sample before and after drying to a constant weight at 105 °C.

Before starting the determination, the weighing vessel with the lid was dried for 30 min in an oven with natural convection Digitheat (J.P. Selecta, Barcelona, Spain) heated to a temperature of 105 ± 2 °C, and then placed in an exicator to cool to room temperature.

The vessel with the lid was weighed on an analytical balance CP224S-OCE (Sartorius, Germany) with an accuracy of 0.0001 g (c), then from an average laboratory sample spent grain, about 5 g was weighed into the vessel with an accuracy of 0.0001 g (a), then they were placed in an oven heated to a temperature of 105 ± 2 °C, the cover was removed, and placed next to the vessel. The sample was dried at 105 ± 2 °C for 3 h. After this time, the vessel with the sample after drying was placed in the exicator until it was cooled to room temperature, and then weighed on an analytical balance with an accuracy of 0.0001 g (b).

Moisture content (M) (%) was calculated from the formula:

(2) $$M=\frac{\left(a-b\right)\cdot 100}{a-c}$$

where:

a—weight of the vessel with the sample before drying, in grams (g),

b—weight of the vessel with sample after drying, in grams (g), and

c—weight of the vessel, in grams (g).

#### 4.2.3. Calculation of Organic Matter Content

The content of organic matter (OM) (%) was calculated from the formula:

OM = 100 − (M + A)(3)

where:

M—moisture of the sample, (%),

A—ash content of the sample, (%).

#### 4.2.4. Determination of Heat of Combustion and Calculation of Calorific Value

Heat of combustion determination was performed using the calorimetric method. The calorific value was calculated according to PN-EN ISO 1928:2020-05 [38]. The method consists of determination of the heat of combustion by total incineration of the sample in an oxygen atmosphere under pressure in the calorimetric bomb placed under water, and measurement of the increase in the temperature of the water.

**Figure 3:**
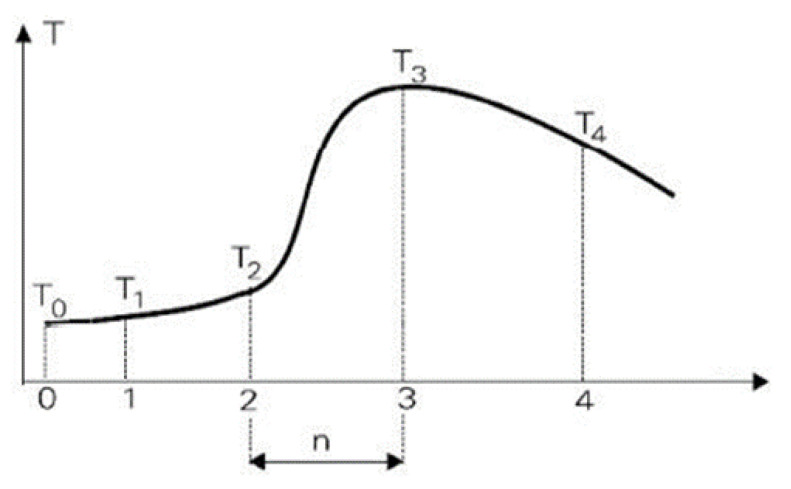
A sample graph of the calorimetric measurement for the calorimeter KL-10.

**Figure 4:**
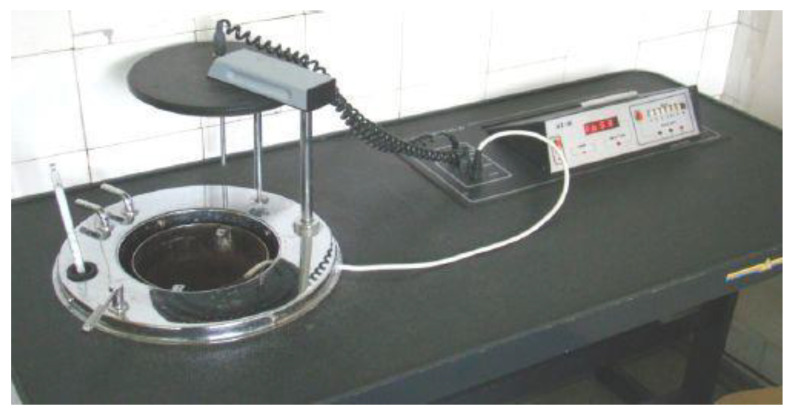
A stand for calorimetric measurements [40].

Approx. 1 g of dry spent grain sample was weighed on the analytic scales with an accuracy of 0.001 g. A pill was prepared from the weighted sample with the help of the press. The sample prepared in this way was placed in the crucible of the calorimetric bomb KL-10 (Figure 3 and Figure 4) (Precyzja-Bit, Bydgoszcz, Poland) and burnt in the oxygen atmosphere under the pressure of 2.8 MPa. For this purpose, the calorimetric bomb with the sample was placed in the vessel filled with water with the capacity of 2.7 L. Resistance wire made from canthal was used to ignite the sample. The heat of combustion was calculated automatically by computer software. The calorimeter measures of the temperatures of the system, which consists of the calorimetric bomb with the sample being combusted and a calorimetric vessel filled with water. 

The calorimeter’s work consists of five cycles:

1. Switching on the device and stabilisation of the temperature inside the calorimeter;
2. Recording the initial temperature *T*1 and measurement of temperature in regular time intervals (every 5 min);
3. Recording temperature *T*2 and ignition of the sample in the calorimeter bomb as well as the measurement of time of combustion of the sample to the maximum temperature of water *T*3;
4. Recording the maximum temperature *T*3 and further measurement of the temperature in regular intervals of time (every 5 min);
5. Recording temperature *T*4, performing computations to obtain the heat of combustion and the calorific value of the examined sample with the use of computer software integrated with the calorimeter, then turning off the device.

The value of the heat of combustion (*Q_s_*) of the examined sample in kilojoules per kilograms (kJ/kg) was determined automatically based on the results of the measurements of characteristic temperatures of the thermal balance of the system using the calorimeter’s computer software according to:

(4) $$Q_{S}=K\cdot (T3-T2-k)$$

where:

*K*—calorimeter’s constant, in kilojoules per kilogram (kJ/kg),

*T*2, *T*3—values of the temperature in measurement points, in Kelvin grades (*K*),

*k*—a correction related to the heat exchange between the calorimeter and the environment, (-), and

*Q_s_—*heat of combustion, in kilojoules per kilogram (kJ/kg).

(5) k=0.5⋅[0.2⋅(T2−T1)+0.2(T4−T3)]+0.2⋅(n−1)⋅(T4−T3)

where:

*T*1 to *T*4—values of the temperature in measurement points, in Kelvin grades (*K*),

*n*—duration of the stage of sample combustion in minutes (measurement from *T*2 to *T*3), and

*k*—correction related to the heat exchange between the calorimeter and the environment, (-).

The results are given as the average values. 

The calorific value (*Q_op_*) of the examined sample in kilojoules per kilogram (kJ/kg) was calculated from the formula:

(6) $$Q_{op}=Q_{s}-24.43\cdot (W+8.94\cdot H)$$

where:

*Q_s_*—heat of combustion, in kilojoules per kilogram (kJ/kg),

*Q_op_*—heat of combustion of the examined sample, in kilojoules per kilogram (kJ/kg),

*W*—water content in the sample (%),

*H*—hydrogen content in the sample (%),

24.43—heat of vaporisation of water at a temperature of 25 °C corresponding to 1% of water in the fuel, and

8.94—conversion factor of the hydrogen content to water.

Based on the data in the literature, Refs. [18,39], it was assumed that the content of hydrogen in BSG amounted to approx. 7.0%.

The calorific value (*Q_opw_*) of the wet sample in kilojoules per kilogram (kJ/kg) was calculated from the formula:

(7) $$Q_{opw}=\frac{100-W_{m}}{100-W}\cdot (Q_{op}+24.43\cdot W)-24.43\cdot W$$

where:

*Q_op_*—calorific value of the dry sample, in kilojoules per kilogram (kJ/kg),

*Q_opw_*—calorific value of the wet sample, in kilojoules per kilogram (kJ/kg),

*W_m_*—water content in the wet sample (%),

*W*—water content in the dry sample (%), and

24.43—heat of vaporisation of water at a temperature of 25 °C corresponding to 1% of water in the fuel.

The heat of combustion and the calorific value were calculated in megajoules per kilogram (MJ/kg).

#### 4.2.5. Determination of Volatile Matter Content

Determination of volatile matter content was performed using the gravimetric method after combustion according to the modification of the method, described by researchers [33,41]. The rule of this method consists of the determination of the mass of the fraction in the sample, which escapes as volatile gases (smoke) during combustion at a temperature of approx. 600 °C.

The crucible was weighed on the analytical scales CP224S-OCE (Sartorius, Germany) with an accuracy of 0.0001 g (c), and 1 g of the sample of dry spent grain was added to the crucible (a). The crucible was placed in the muffle furnace for combustion SM-2002 (Czylok, Jastrzębie-Zdrój, Poland), heated to a temperature of approx. 600 ± 15 °C, and combusted for approx. 10 min. During the process, smoke coming out of the opening in the upper part of the muffle furnace was observed. The crucible with the desmoked sample was then taken out of the furnace with the help of metal pliers and placed in the exicator. After cooling, the crucible with the desmoked residue was weighed on the analytic scales with an accuracy of 0.0001 g (b). The determination was conducted in two replicates.

Volatile matter content (VM) (%) was calculated from the formula:

(8) $$\mathrm{VM}=\frac{\left[\left(a-c\right)-\left(b-c\right)\right]\cdot 100}{a-c}$$

where:

a—mass of the crucible with the sample prior to combustion, in grams (g),

b—mass of the crucible with the residue after combustion, in grams (g), and

c—mass of the crucible, in grams (g).

#### 4.2.6. Calculation of Fixed Carbon Content

Fixed carbon is the solid combustible residue that remains after a biomass particle is heated and the volatile matter is expelled. The fixed carbon content of a biomass is determined by subtracting the percentages of moisture, volatile matter, and ash from a sample [41].

The content of fixed carbon (FC) (%) was calculated from the formula:

FC = 100 − (M + A + VM)(9)

where:

M—moisture content in the sample, (%),

A—ash content in the sample, (%), and

VM—volatile matter content in the sample, (%).

#### 4.2.7. Statistical Analysis

Determinations were conducted in three replicates (n = 3). The results are given as the average values with standard deviation (±SD). 

Homogeneity of variance in groups BM, BM + B, and BM + WM were tested by Levene’s test. We used Welch’s ANOVA in the case of lack homogeneity of variance, and one-way ANOVA otherwise. Multiple mean comparisons were performed according to the Tukey procedure. Statistical analyses were carried out on the significance level of alpha 0.05. Means on graphs are presented as mean ± SE. Since BSG was characterised by several features, we aimed to investigate an overall effect of additions on these features. For this reason, multidimensional space was reduced to two latent variables obtained by principle component analysis.

## 5. Results 

The study involved three different types of brewer’s spent grain (BM, BM + B, and BM + WM), that are different in ingredient composition and moisture.

### 5.1. Chemical Composition of Spent Grain

Ash and water contents were determined in the samples of wet spent grain. Determinations were also conducted for samples of dry spent grain. For this purpose, average laboratory samples were prepared for each type of spent grain. An average laboratory sample was dried at a temperature of approx. 60 °C to a moisture of approx. 4.5%. The average sample was mixed carefully, and size-reduced by trituration in the porcelain mortar. 

Table 1 presents the results of the content of the examined components for wet spent grain samples. 

Ash content in samples of wet spent grain with different ingredient composition ranged between 0.8 and 1.0%. The analysed BSG samples had a moisture content from 77.81 to 79.71% (average approx. 79.1%). Table 2 contains the composition of dry brewer’s spent grain. The method of preparation of samples is described above. 

The examined samples of spent grain contained between 3.80 and 4.09% of ash. The sample of spent grain with the addition of barley had the lowest ash content (BM + B) in comparison with the other samples, whereas the sample of barley malt (BM) had the highest ash content. The moisture content of the spent grains after drying at a temperature of approx. 60 °C ranged from 4.17 to 4.90 (average approx. 4.47%).

The differences in the ash content between individual samples of spent grain amounted to 0.3% d.m.

### 5.2. Energy Properties of Spent Grain

The calorific value was determined based on the calorimetric method that specifies the usefulness of the material being examined for energy purposes. Examinations of the samples of spent grain were performed for the average laboratory samples, which were dried at a temperature of the drying air of approx. 60 °C. The preparation of the samples is described above. 

Table 3 contains basic data specifying energy properties of brewer’s spent grain (BSG) in the dry form. The calorific value of wet spent grain was calculated on the basis of the determined moisture content of fresh and dried samples.

The assessed samples of spent grain were characterised by extremely similar contents of organic substances, which amounted to 91.0–91.9% by weight (95.7–96.0% d.m.). Ash and water present in spent grain do not have any energy value, and thus are so-called ballast, i.e., they are undesirable in the fuel. 

The examined samples of spent grain were characterised by similar contents of volatile matter, which escaped in the form of smoke and steam. The determined content was between 77.6 and 78.7%. Estimates of the content of fixed carbon fluctuated from 13.17% for BSG from a mixture of barley and wheat malts (BM + WM) to 14.26% in the case of spent grain barley malt mixed with malting barley (BM + B). The heat of combustion of the spent grain in the calorimetric bomb was at the level of 17.27–17.49 MJ/kg, and the calculated calorific value ranged from 15.63 MJ/kg for spent grain from barley malt with barley (BM + B) to 15.86 MJ/kg for spent grain from barley malt and wheat malt (BM + WM). In turn, the calorific value calculated for wet malt was from 1.42 MJ/kg for the sample from barley malt and wheat malt (BM + WM) to 2.01 MJ/kg for the sample from barley malt (BM). 

**Figure 5:**
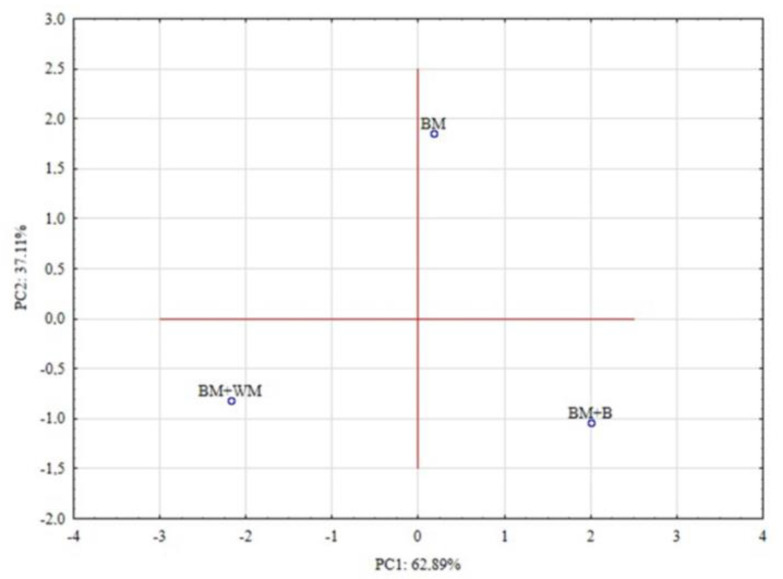
PCA plot of analysed observations.

PCA resulted in extraction of two eigenvalues which corresponded to the first and the second principal component and explained, respectively, 62.89% and 37.11% of variability (Figure 5).

Then, principal components were interpreted on the basis of their correlations with original variables. PC1 correlated strongly with VM, FC, HC, and CV, which suggested that this component may be interpreted as the energy value of BSG, whereas PC2 correlated highly with three other variables (M, A, and OM), which indicated that PC2 is rather related to mass potential (Table 4).

**Figure 6:**
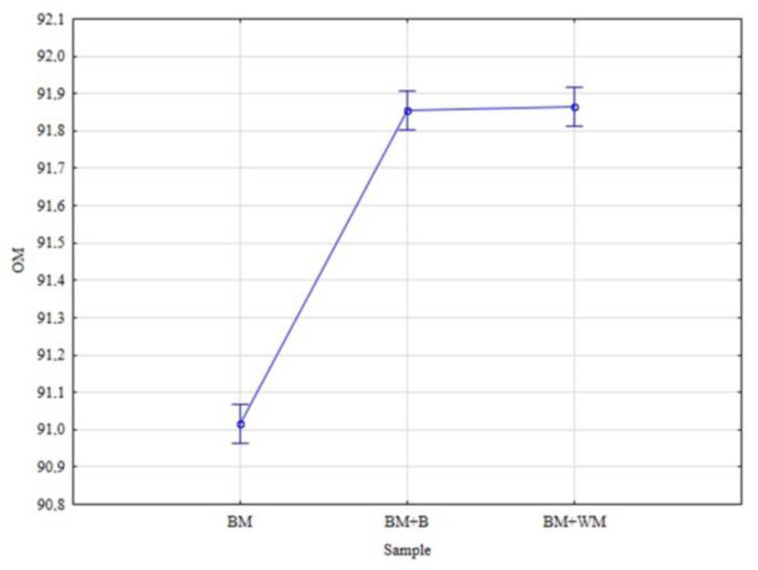
Mean contents of organic matter (OM) in BSG.

The analysis conducted for moist spent grains revealed that malt additions significantly modified ash content and organic matter in BSG. In the case of VM, FC, HC, and CV, no significant differences between investigated BSGs were observed (Figure 6). 

**Figure 7:**
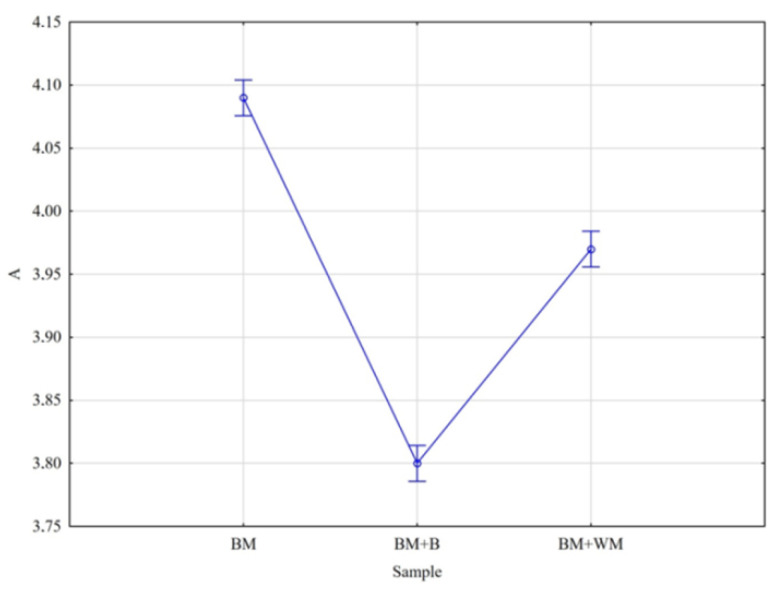
Mean contents of organic matter (A) in BSG.

For ash, significant differences were observed between all factor levels contrary to organic matter where difference between means calculated for BM + B and BM + WM were negligible (Figure 7).

**Figure 8:**
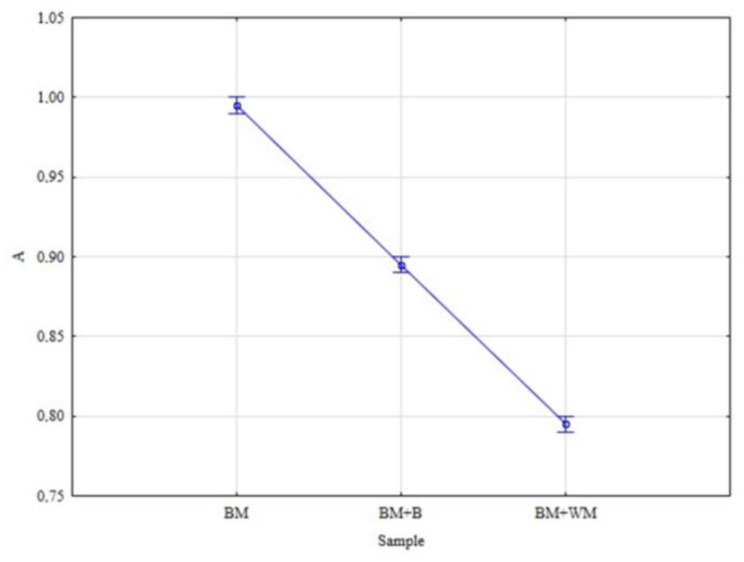
Mean contents of ash (A) in BSG.

**Figure 9:**
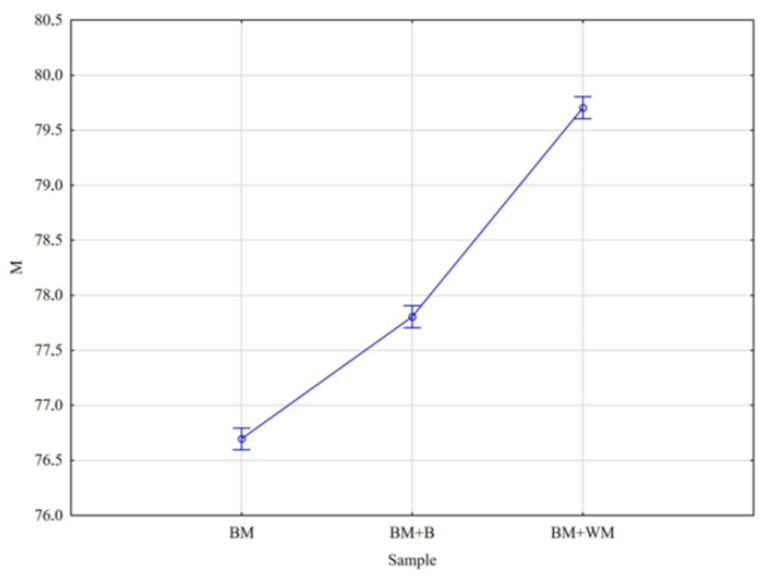
Mean contents of ash (M) in BSG.

The analysis of data for dry spent grains showed that the effect of malt additions on the moisture content as well as on the ash content in BSG was significant. For both variables (Figure 8 and Figure 9), significant differences were observed between BM and BM + B and between BM + B and BM + WM.

## 6. Discussion

Biomass is considered the third largest and most available source of renewable energy worldwide. In Poland, it is used for energy purposes by direct combustion (e.g., straw, wood, energy plants, and cereal grain) or processed into liquid biofuels (bioethanol) and gaseous biofuels (biogas) [30,31]. The determinants of the calorific value of biomass are its moisture and ash content. 

Brewer’s spent grain, which is a waste product left over from beer production, is a plant-derived raw material, which, like other types of biomass, might be used as an alternative source of renewable energy [42]. 

### 6.1. Composition of Spent Grain

After wort separation, spent grain has a relatively high water content (approx. 75–85%) [2,5,20,27,39]. The obtained results of the analysed samples of BSG contained on average approx. 79.1% of water and are consistent with the literature data. Refs. [2,3,5,12] have reported that brewer’s spent grain (BSG), apart from water, mainly contains non-starch polysaccharides, such as hemicelluloses (approx. 19–42% d.m.), cellulose (approx. 0.3–40% d.m.), and lignin (approx. 9.2–28% d.m.), as well as starch (approx. 1–12% d.m.), protein (approx. 14–31% d.m.), lipids (approx. 3–13% d.m.), ash (approx. 1–5% d.m.), and trace amounts of other substances of the extract. The chemical composition of spent grain may differ depending on the type and quality of the batch of raw material for wort production as well as malting technology and malt filtration. 

Water and ash, which are non-combustible components and lower the calorific value of the material, determine the usefulness of biomass as biofuel, while organic substances in the material such as fat, carbohydrates, including fibre (hemicellulose, cellulose, and lignin), and protein are combustible and supply energy during combustion. Protein contained in the spent grain, apart from carbon, oxygen, and hydrogen, contains small amounts of nitrogen (up to approx. 4.5%) and sulphur (up to approx. 0.1%) according to Refs. [19,20], which slightly reduce the energy efficiency of biomass. 

Ash content determined in the samples of wet spent grain from different ingredient inputs ranged from 0.8 to 1.0% (approx. 0.9%). The organic substance content in the analysed samples of wet spent grain ranged from 19.5% (BM + WM) to 22.3% (BM), i.e., it should be assumed that organic matter amounts are approx. 1/5 of the raw material mass, or approx. 20% of wet spent grain. The analysis of spent grain composition with different ingredient inputs, after conversion to dry mass, showed that the examined samples contained 4.0–4.3% d.m. of ash (averagely approx. 4.1% d.m.). The remaining part of the dry matter of the assessed BSG samples consisted of organic matter (up to approx. 96% d.m.). Similar values have been given by other researchers [3,5,20,21,27].

### 6.2. Dried Spent Grain

Results of the chemical composition of average samples of brewer’s spent grain after drying are presented in Table 2. The chemical composition of dry samples of brewer’s spent grain was similar to the values obtained in the dry substance, since as a result of drying, the dry substance of the samples ranged from 95.1% for spent grain from barley malt (BM) to 95.8% for spent grain from barley malt and wheat malt (BM + WM). The determined value of ash in the analysed samples ranged from 3.8% (BM + B) to 4.1% (BM). These values are similar to the ash content determined in bran (approx. 5%) and higher than that in barley grain (approx. 3%) and wheat grain (approx. 2%) [43]. Additionally, higher ash content for samples of BSG was obtained by other researchers, e.g., the ash content in spent grain from malted sorghum was approx. 7.1% [33], and from barley malt (examinations conducted in Mexico)—6.4% [19].

### 6.3. Fuel Properties

In order to characterise fuel properties of the samples of brewer’s spent grain, average samples of spent grain dried at a temperature of the drying air of approx. 60 °C were used for examinations. Table 3 contains the results of examinations of the types of brewer’s spent grain assessed as biomass for energy purposes. 

According to the literature [20,21,34,44], in the next few years, plant-derived biomass may become one of the main renewable resources of electrical and heat energy, which may result in numerous benefits such as the reduction in greenhouse gas emissions, increase in employment, or reduction in expenditure on fossil fuel exports, e.g., crude oil or natural gas. 

The calorific value is one of the most important parameters characterising solid fuels. The energy value of all types of biomass, including spent grain, depends on the moisture. Water content is extremely closely correlated with the calorific value. The higher the content of water in the material, the lower its calorific value [20,21,43,44,45].

Organic matter in spent grain or in other types of biomass is combusted. It is made of compounds that contain carbon and other combustible elements. Non-combustible substances in the raw material such as water and ash are so-called fuel ballast and decrease its gross calorific value. In turn, volatile matter is a product of combustion, and is released in the form of smoke, flammable gases, and steam in certain conditions.

Samples of brewer’s spent grain as the object of the research had similar organic matter contents, which may indicate that approx. 91–92% of spent grain components may be combusted to obtain energy under appropriate conditions. The determined amount of volatile matter in spent grain samples was between 77.6% and 78.7%. Similar values (approx. 78.5–79.9%) were obtained by Mexican researchers [19,20], who examined energy properties of two types of biomass left over from beer production (BSG) and tequila (agave fibre). A considerably lower content of volatile matter, at the level of 47%, was obtained from wheat spent grain [33]. The obtained result may be attributed to the type of grain used or different methodology applied by the researchers. 

The content of fixed carbon in the analysed BSG samples was in the range 13.17–14.26% (average approx. 13.6%). BSG analysed by Bieniek et al. [23] contained lower amounts of fixed carbon—8.85%, and the samples tested by Coronado et al. [20] contained bound carbon at the level of 17.48%. A high content of bound carbon and volatile substances increase the calorific value of all biomass fuels [19,21,33].

The heading value of BSG determined in the calorimetric bomb was similar and amounted from approx. 17.3 MJ/kg for spent grain from barley malt with the addition of barley (BM + B) to approx. 17.5 MJ/kg for spent grain from barley and wheat malts (BM + WM). Extremely similar values of the heat of combustion were obtained by Żabiński et al. [40] for wheat and triticale quality grain (approx. 17.3 MJ/kg), while the heat of combustion determined for barley grain was approx. 17.6–17.8 MJ/kg. The spent grains tested by Coronado et al. [20] from the process in which 85% of two-row malts and 15% of special malts were used, characterised the calorific value at the level of 18.7 MJ/kg. The obtained results of the analysis of spent grain from the brewing craft industry may indicate a lower degree of extraction of soluble sugars and nitrogen compounds into the wort from special malts (15% of the raw material input) during mashing or lautering of the BSG with water.

The calorific value of the examined types of spent grain in dry samples (moisture approx. 4.5%) ranged from 15.6 MJ/kg for spent grain from barley malt with barley (BM + B) to 15.9 MJ/kg for spent grain from barley and wheat malts (BM + WM). Higher calorific values of spent grain from barley malt were obtained by Liñán-Montes et al. [19] and Weger et al. [3], which were approx. 19.06 MJ/kg and approx. 20.6 MJ/kg, respectively. Spent grain examined by Manyuchi and Frank [33] had a lower calorific value (approx. 12.6 MJ/kg). The data given by Niedziółka and Zuchniarz [44] indicate that rape straw (15.0 MJ/kg), wood dust (15.2–20.1 MJ/kg), or barley straw (16.1 MJ/kg) had similar calorific values to the types of BSG examined. In turn, Kaszkowiak and Kaszkowiak [46] examined barley harvested in two successive years and demonstrated that the calorific value of barley grain ranged from approx. 16.7 MJ/kg to approx. 17.2 MJ/kg. Examinations of selected types of biomass conducted by Mółka and Łapczyńska-Kordon [43] demonstrated a low calorific value of bran (above 14 MJ/kg) in comparison with beech (above 20 MJ/kg). The calorific value of cereals ranged from 15.3 MJ/kg for barley to 16.6 MJ/kg for oat. In the studies by Kosakowski et al. [22] the calorific value of BSG was 18.9 MJ/kg, while in other analysed types of biomass, it ranged from 14.9 MJ/kg (flavoured spirit production waste, i.e., lime, grapefruit, and lemon) to 19.8 MJ/kg (apple pomace).

The calorific value of cones and wood (18.1–19.9 MJ/kg) [45] or wood briquettes (16.9–20.4 MJ/kg) [44] was higher than the calorific value of the assessed samples of BSG. Solid fossil fuels have much higher calorific values. According to the information provided by Porowski [47], the calorific value in the air-dry basis was: 19.7–23.2 MJ/kg for brown coal, 28.6 MJ/kg for charcoal, 29.4–32.3 MJ/kg for hard coal, 31.1 MJ/kg for anthracite, and 14.5 MJ/kg for peat.

The obtained results of the calorific value of spent grain with a moisture of approx. 79% (1.4–2.0 MJ/kg) indicated that it may not be used for energy purposes. Therefore, dewatering of spent grain is highly recommended. 

To summarise, it could be concluded that the analysed samples of brewer’s spent grain, irrespective of the type of raw material used for wort production, demonstrated similar parameters significant for the calorific value. 

## 7. Summary and Conclusions

Biomass has a great energy potential that is now used all over the world. In the near future, biomass may become one of the main renewable energy sources in Poland by forecasts. 

BSG is a waste of plant origin from the production of beer. This type of biomass is suitable for energy use. A high water content (approx. 80%) should be considered as a factor limiting the industrial use of BSG. 

The analysis of the current state of knowledge on the energetic use of BSG and research on the fuel properties of grain spent from various raw materials allowed for the evaluation of this waste product as an alternative source of renewable energy.

Energy from BSG has long been used to meet the energy needs of the brewery in countries closer to the equator and in Western Europe (e.g., Germany). In Poland, this material is rarely used, most often in an agricultural biogas plant. BSG usually undergoes mechanical pre-treatment, which involves dewatering. Lowering the moisture content in spent grain by drying is energy-consuming and less cost-effective from an economic point of view. Most biomass of plant origin has a moisture content above 15%. Therefore, drying is usually the only method of making biomass suitable for energy purposes.

Based on the obtained results of the research and literature review related to the use of brewer’s spent grain (BSG) for energy purposes, it was possible to formulate the following conclusions:

1. Ash content determined in the examined samples of dry spent grain was similar (approx. 3.8–4.1%). No differences between the examined samples of spent grain were observed regarding organic substance content (approx. 91.0–91.9%);
2. Volatile matter content in dry spent grain was similar for all the analysed samples and ranged from 77.6 to 78.7%;
3. Dried spent grain from barley and wheat malt had the highest heat of combustion—approx. 17.49 MJ/kg, and dried barley malt with the addition of barley had the lowest heat of combustion—approx. 17.27 MJ/kg (the difference was approx. 0.22 MJ/kg);
4. BSG, both wet and dry with different ingredient inputs, demonstrated extremely similar parameters significant for the calorific value of this raw material. The calorific value determined for dry spent grain was extremely similar and ranged between approx. 15.63 and 15.86 MJ/kg. The obtained values were comparable to the calorific value of peat and other types of biomass, e.g., cereal grain, rape and barley straw, or wood dust.
5. The estimated calorific value for the examined samples (approx. 1.42–2.01 MJ/kg) indicated that it is necessary to dewater biomass to improve its energy properties;
6. In practice, pre-treatment of brewer’s spent grain involves mechanical pressing or convective drying of biomass to lower the moisture to a certain level, which makes its combustion in biomass boilers or other similar devices possible. Following mechanical pre-treatment or enzymatic hydrolysis, brewer’s spent grain is used for the production of biogas and bioethanol in numerous countries. Moreover, its availability and low cost make this type of biomass promising as a raw material to obtain energy from renewable resources.

## Figures and Tables

**Table 1:** Content of components in wet spent grain.

Parameters	Type of Wet Brewer’s Spent Grain (BSG)		
	BM	BM + B	BM + WM
Moisture, %	79.70 ± 0.08	77.81 ± 0.11	79.71 ± 0.21
Total ash, %	1.00 ±0.01	0.90 ± 0.01	0.80 ± 0.01

**Table 2:** Composition of samples of dry spent grain.

Parameters	Type of Dry Brewer’s Spent Grain (BSG)		
	BM	BM + B	BM + WM
Moisture, %	4.90 ± 0.02	4.35 ± 0.11	4.17 ± 0.05
Total ash, %	4.09 ± 0.01	3.80 ± 0.01	3.97 ± 0.03

**Table 3:** Calorific value and other energy parameters of brewer’s spent grain (dry basis).

Heat Properties Parameters	Type of Brewer’s Spent Grain (BSG)		
	BM	BM + B	BM + WM
Organic matter, %	91.02 ± 0.04	91.86 ± 0.12	91.87 ± 0.2
Volatile matter, %	77.70 ± 1.41	77.60 ± 1.27	78.70 ± 0.28
Fixed carbon, %	13.22 ± 1.38	14.26 ± 1.15	13.17 ± 0.26
Heat of combustion, MJ/kg	17.38 ± 0.31	17.27 ± 0.22	17.49 ± 0.19
Calorific value, MJ/kg	15.73 ± 0.36	15.63 ± 0.37	15.86 ± 0.40
Calorific value of wet spent grain, MJ/kg	2.01	1.76	1.42

**Table 4:** Correlations of PCs and original variables.

Variable	PC1	PC2
M	−0.67854	**−0.73456**
A	−0.52356	**0.851988**
OM	−0.08195	**−0.99664**
VM	**−0.9325**	−0.36117
FC	**0.892429**	−0.45119
HC	**−0.99742**	0.071727
CV	**−0.99999**	−0.00338

## Data Availability

The data presented in this study are available upon request from the corresponding author.
